# Unexpected Transient Glioblastoma Regression in a Patient Previously Treated with Bacillus Calmette–Guérin Therapy: A Case Report and Immunomodulatory Effects Hypothesis

**DOI:** 10.3390/jpm13121661

**Published:** 2023-11-28

**Authors:** Gianluca Scalia, Gianluca Ferini, Salvatore Marrone, Maurizio Salvati, Vicky Yamamoto, Babak Kateb, Reinhard Schulte, Stefano Forte, Giuseppe Emmanuele Umana

**Affiliations:** 1 Neurosurgery Unit, Department of Head and Neck Surgery, Garibaldi Hospital, 95123 Catania, Italy; 2 Department of Radiation Oncology, Istituto Oncologico del Mediterraneo, 95029 Viagrande, Italy; gianluca.ferini@grupposamed.com; 3 Neurosurgical Clinic, AOUP “Paolo Giaccone”, Post Graduate Residency Program in Neurologic Surgery, Department of Biomedicine Neurosciences and Advanced Diagnostics, School of Medicine, University of Palermo, 90127 Palermo, Italy; salvo.mr89@gmail.com; 4 Department of Neurosurgery, Policlinico “Tor Vergata”, University of Rome “Tor Vergata”, 00133 Rome, Italy; salvati.maurizio@libero.it; 5 University of Southern California-Norris Comprehensive Cancer Center, Los Angeles, CA 90033, USA; vyamamot@usc.edu; 6 Brain Mapping Foundation, Los Angeles, CA 90272, USA; babak.kateb@worldbrainmapping.org; 7 School of Medicine, Loma Linda University, Loma Linda, CA 11085, USA; schulte@llu.edu; 8 Genomics and Experimental Oncology Unit, Istituto Oncologico del Mediterraneo, 95029 Viagrande, Catania, Italy; stefano.forte@grupposamed.com; 9 Department of Neurosurgery, Gamma Knife and Trauma Center, Cannizzaro Hospital, 95126 Catania, Italy; umana.nch@gmail.com

**Keywords:** glioblastoma, Bacillus Calmette–Guérin, bladder cancer, regression, immunomodulatory effects, case report

## Abstract

(1) Background: Glioblastoma multiforme (GBM) is a highly aggressive brain tumor with limited treatment options and poor prognosis. Bacillus Calmette–Guérin (BCG), a live attenuated strain of Mycobacterium bovis, has been used as an immunotherapeutic agent in bladder cancer and has shown non-specific beneficial effects. This report presents a unique case of GBM regression following BCG therapy for bladder cancer, suggesting the potential systemic immunomodulatory effects of BCG on GBM. (2) Case Presentation: A 67-year-old male with a history of bladder cancer treated with BCG presented with neurological symptoms. Imaging revealed two GBM lesions, and surgery was performed to remove one. Subsequently, the patient experienced complete tumor regression after initial stability. (3) Conclusions: This case highlights the potential of BCG or other immunotherapies in GBM treatment and underscores the need for further research. Understanding the immunomodulatory effects of BCG on GBM could lead to innovative therapies for this devastating disease; although, overcoming the immune evasion mechanisms in the brain is a significant challenge. Further investigation is warranted to explore this promising avenue of research.

## 1. Introduction

Glioblastoma multiforme (GBM) stands as the most prevalent primary malignant brain tumor in adults, characterized by its aggressive growth pattern and resistance to treatment. Despite standardized treatment protocols involving maximal safe surgical removal, followed by radiotherapy and chemotherapy utilizing temozolomide, the median survival rate for patients remains disappointingly low. Consequently, there exists an urgent demand for more effective therapies targeting GBM. Bacillus Calmette–Guérin (BCG), a live attenuated strain of Mycobacterium bovis, has a long history of use as a vaccine for tuberculosis and as an immunotherapeutic agent in bladder cancer treatment. The BCG vaccine has shown non-specific beneficial effects, reducing morbidity and mortality from various types of infections and potentially some malignancies [1,2]. In bladder cancer, BCG therapy induces a potent local immune response leading to the destruction of cancer cells [3,4]. This report presents a unique case in which a patient, previously treated with BCG for bladder cancer, subsequently developed GBM, which then regressed completely. This occurrence raises the possibility of systemic immunomodulatory effects induced by BCG influencing the course of GBM.

## 2. Case Presentation

A 67-year-old male was referred to our emergency department due to the progressive deterioration of his consciousness. His previous medical history was positive for bladder cancer diagnosed 6 years earlier, and he had been treated for 5 years with topical bladder BCG instillations. The patient also had a history of arterial hypertension.

### 2.1. Clinical Examination and Findings

The clinical examination revealed a Glasgow Coma Scale (GCS) score of 10/15 and left-sided hemianopia. A detailed physical examination was impossible due to the poor neurological condition of the patient.

### 2.2. Imaging and Initial Diagnosis

**Figure 1:**
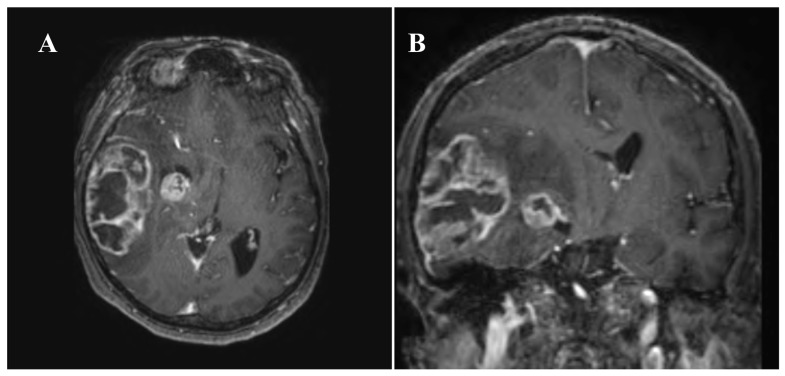
Preoperative T1-weighted brain MRI axial (**A**) and coronal (**B**) sequences with gadolinium reveal the presence of lesions with irregular margins, containing diffuse necrotic and hemorrhagic/hemosiderin-rich intralesional components. It exhibits pathological and heterogeneous contrast enhancement, localized in the right lateral temporal region. This lesion measures approximately 70 mm antero-posteriorly, 40 mm medio-laterally, and 56 mm cranio-caudally. It is associated with a substantial perifocal edema extending into the temporal, deep frontoparietal, and ipsilateral nucleus-capsule. Another smaller lesion of similar significance and characteristics, with a rounded morphology, is observed in the ipsilateral temporo-mesial region.

A head CT scan showed two lesions: the largest located to the right, temporally and posteriorly, and a smaller one positioned medially and anteriorly to it. The midline shift was approximately 12 mm. The patient underwent an urgent brain MRI with Gadolinium to better characterize the two lesions, measuring 70 × 40 mm^2^ and 17 × 18 mm^2^, both associated with peritumoral edema and central necrosis (Figure 1).

### 2.3. Further Diagnostic Tests

The patient also underwent a PET-FDG total body scan and a CT scan with contrast enhancement, which excluded systemic lesions.

### 2.4. Initial Surgical Intervention

**Figure 2:**
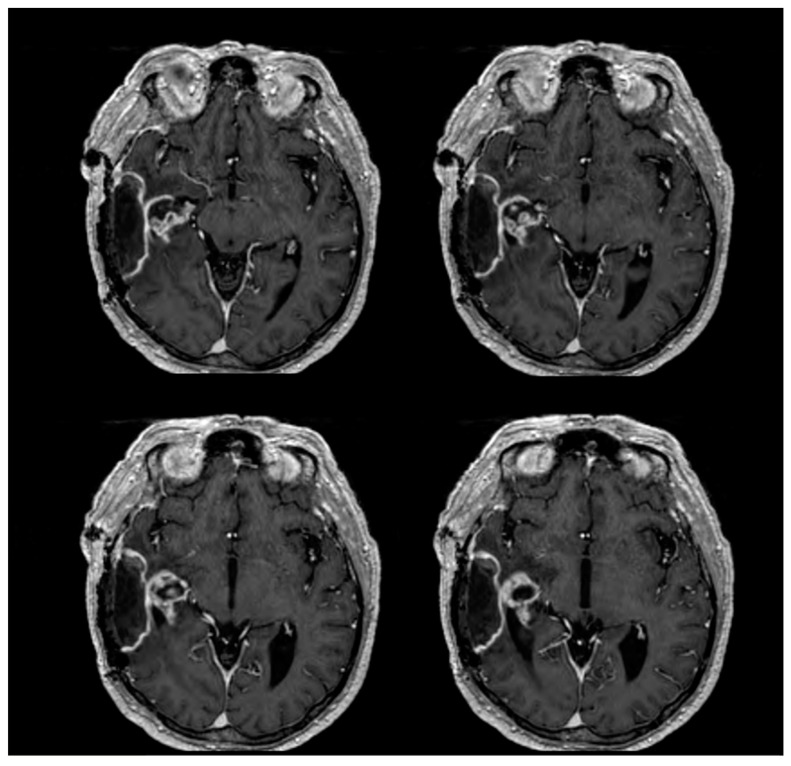
Postoperative T1-weighted brain MRI axial sequences with gadolinium demonstrate the complete removal of the larger lesion and the stability of the smaller temporo-mesial right lesion.

An urgent right temporal craniotomy was performed to remove the larger lesion, but the procedure was quickly stopped due to intraoperative seizures, and the smaller lesion was left in place. The postoperative period was uneventful, and the patient progressively fully recovered. Postoperative imaging showed complete removal of the larger lesion and the stability of the smaller one (Figure 2).

### 2.5. Histological Examination

Histological examination documented the lesion as a high-grade glial tumor, wildtype IDH, with extensive necrosis and microvascular proliferation consistent with glioblastoma (WHO grade IV). MGMT methylation was observed (32%).

### 2.6. Treatment and Follow-Up

**Figure 3:**
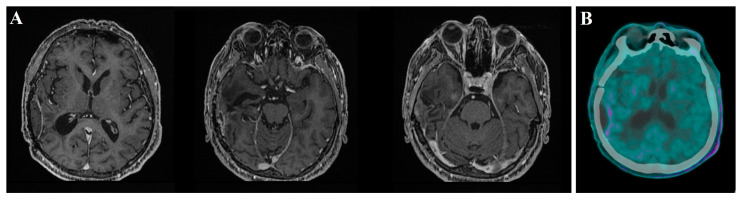
One-year follow-up postoperative T1-weighted brain MRI axial sequences with gadolinium reveal locoregional meningeal enhancement in the absence of neoangiogenesis signs on perfusion study. The previously observed right temporo-mesial lesion shows no enhancement and exhibits no signs of neoangiogenesis in the perfusion study (**A**). The 11C-methionine PET/CT brain examination documents a mild, heterogeneous tracer accumulation along the right temporo-parietal surgical cavity margin, with no additional abnormalities or asymmetries detected in the remaining brain areas. This is indicative of the absence of cerebral disease (**B**).

Post-operatively, the patient was prescribed levetiracetam at a dosage of 500 mg twice daily for a duration of 4 weeks to manage the risk of seizures. Additionally, a course of Dexamethasone, initially administered at 4 mg twice daily for 15 days, was gradually tapered down to mitigate inflammation and cerebral edema caused by the surgery. The patient started the Stupp protocol and underwent serial MRI scans with Gadolinium every 3 months. During follow-up, tumor stability was observed for about 1 year. Afterward, MRI scans documented no recurrence in the operative field and complete tumor regression of the unresected lesion. Serial MRI scans with Gadolinium, spectroscopy, perfusion studies, and PET-met confirmed the regression of the glioma (Figure 3).

### 2.7. Disease Progression

**Figure 4:**
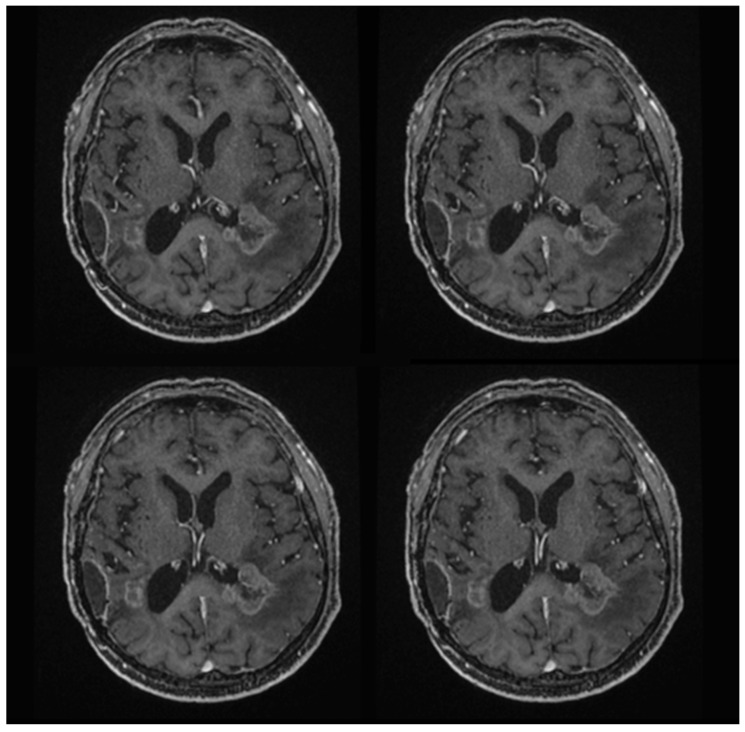
T1-weighted postoperative brain MRI sequences at more than one year of follow-up reveal two new brain lesions: one in the right para-trigonal area and another infiltrating the splenium of the corpus callosum on the left side. There is no evidence of recurrence of the previous lesions on the right.

The patient appeared to be tumor-free for almost 1 year, but 21 months after surgery he developed a recurrence of bladder cancer, and BCG therapy was administered. After 1 month, two new brain lesions appeared: one in the right paratrigonal area and another infiltrating the splenium of the corpus callosum on the left side; no recurrence of the original lesions on the right was detected (Figure 4).

### 2.8. End-of-Life Care

It was decided to withdraw the patient from any treatment due to the severity of the tumor spread. The patient died a few days later, 22 months after surgery.

## 3. Discussion

BCG, an attenuated microorganism derived from Mycobacterium bovis, becomes avirulent after undergoing several laboratory processes. Its capacity to stimulate an effective immune response makes it valuable in vaccinations against tuberculosis and leprosy in high-endemic areas. It is also utilized in neurology for the immunomodulation treatment of multiple sclerosis and in oncology [4].

In urology, it has been used towards non-muscle-invasive bladder cancer, resulting in reduced recurrence and progression. This is achieved by stimulating in situ innate and acquired immunity, thereby providing focused control of tumor expansion [1,2]. Additionally, BCG can directly induce tumor necrosis through free radical-mediated oxidative stress, boosting the immune response, or in certain cases, causing controlled cell death or apoptosis [3].

Recent studies have shown that neoplastic cells can phagocytize the bacillus through endocytosis, which, once internalized, activates intrinsic caspase-dependent pathways aimed at cellular death, facilitating a cytoreductive effect [4]. This internalization does not occur in healthy urothelium cells. Despite the stronger capacity in less differentiated neoplastic cells, there are evident cases of under-response or non-responsiveness to the bacillus [5]. This is likely due to the bacillus’s paradoxical ability to inhibit other cytokine-mediated cytotoxic signals intended to ensure neoplastic cell death [6]. As a result, both the cytotoxic and immunomodulatory effects of BCG continue to be investigated through in vitro and in vivo experiments.

GBM is a neurosurgical disease with a poor prognosis. However, advancements in chemotherapy and radiotherapy, such as the Stupp protocol, have increased survival from six months to two years for patients with GBM IDH-mutant [7].

Rare cases of pathology regression have been reported, where off-label usage of drugs for vasogenic edema (such as dexamethasone) is hypothesized to inhibit inflammation mediators like metalloproteinase and adrenomedullin. These factors affect neo-angiogenesis underlying tumor proliferation and its expansion [8]. Corticosteroids, along with antiepileptic drugs (levetiracetam, valproate), are believed to increase neoplastic cells’ chemotherapy sensitivity by interfering with the activity of specific D cyclins involved in stem cell transformation [9]. Indeed, a unique case of the regression of a glioblastoma following therapy with dexamethasone and levetiracetam has already been reported [10]. Levetiracetam has also been invoked as a trigger for unexpected, likely immune-related events in patients previously submitted to radiotherapy [11]. Several studies have proven that such an antiepileptic drug may act as a sensitizer of concurrent chemoradiotherapy in glioblastoma, enhancing its antitumor efficacy and, ultimately, prolonging patients’ survival [12,13,14,15]. An almost complete regression of a recurrent anaplastic oligodendroglioma was reported in a patient treated with levetiracetam in addition to temozolomide and dexamethasone [16].

In the 1970s, Albright demonstrated a hypersensitivity reaction in GBM patients after BCG inoculation [17], and in 1985, Knerich et al. used BCG as immunotherapy in combination with chemotherapy for brain tumors (both primary and metastatic) [18]. Current research is focusing on testing vaccines capable of immunizing the body against specific molecular receptors on the membrane of neoplastic cells.

The use of bacterial microorganisms like Vibrio cholerae for pre-immunizing mice with anaplastic ependymoblastomas appears to stimulate an immune response that interferes with neoplastic proliferation and increases median survival [19].

Specific antibodies against tumor cells were extracted from Macaca Fascicularis primates’ serum after being inoculated with BCG and human glioma cell cultures. This extraction confirmed the effectiveness and non-toxicity of the serum, as it stimulated a hyperimmune response without attacking the autologous tissues (encephalomyelitis) [20].

However, there are limited studies explaining the immunotherapeutic function of BCG in human glioma and its potential role in tumor regression. The development of hybrid vaccines, which combine the microbiological component BCG with autologous neoplastic cells that have lost their ability to divide due to radiation but can still be processed and recognized by the immune system, has made significant progress in research [21]. However, there are still many unanswered questions about the mechanism of action and efficacy of this approach in tumor immunobiology.

The mechanisms by which BCG achieves this involve both innate and adaptive immunity. BCG is known to stimulate toll-like receptor 2 (TLR2), leading to the activation of the NF-κB pathway and the subsequent release of pro-inflammatory cytokines such as TNF-α, IL-1, IL-6, and IL-12. Furthermore, BCG stimulates the maturation of dendritic cells, enhances the cytotoxicity of natural killer (NK) cells, and promotes the activation and proliferation of both CD4+ and CD8+ T cells [22].

The possibility of BCG having systemic immunomodulatory effects that could influence the course of other malignancies, including GBM, is intriguing. The regression of GBM in this case could be hypothesized to result from an enhanced systemic immune response induced by BCG, possibly involving the activation of NK cells or cytotoxic T cells against GBM cells, or the production of cytokines that inhibit tumor growth or promote apoptosis of cancer cells.

However, it is also possible that the observed regression of GBM in this case is coincidental and unrelated to BCG therapy. Besides the above-mentioned possible role of dexamethasone and levetiracetam, the components of STUPP protocol may also have been a determinant in achieving the observed tumor disappearance: the event of complete response to chemoradiotherapy or temozolomide alone occurred rarely [23,24]. Given the rarity of spontaneous regression in GBM, which was reported as transient (like in our case) in only two other patients [25,26], the temporal relationship to BCG therapy suggests a possible causal relationship, but this cannot be confirmed based on a single case. Furthermore, bladder cancer recurrence followed by the development of two new lesions described here might be related to each other. One hypothesis could be that the bladder recurrence was the expression of the BCG-related lack of immune coverage, leading to both recurrences. However, even surgery could be added to the list of the above determinant factors, lying as a further confounder behind the cause of the observed result. Indeed, surgical procedures (intended both as biopsy and resection of the primary tumor or metastases) may elicit immunologic surveillance through proinflammatory injuries leading up to complete regression of any residual macroscopic distant tumor [27]. This very rare phenomenon has been reported for a variety of tumor histologies, although not yet for gliomas [25,26,28,29,30].

A comprehensive theory would bring into play all the afore-mentioned factors: it is possible that the subtotal resection, functioning as an immune un-blocker thanks to its association with radiotherapy, temozolomide, levetiracetam, and dexamethasone, may have facilitated the host immune system to cross-react against the residual glioblastoma cells after a long-standing stimulation with BCG. The latter, once stopped for several months, might have been insufficient to imprint an effective immunological memory to prevent and contrast the subsequent bladder and brain recurrences. Further studies, including preclinical studies in animal models and clinical trials in patients, are needed to investigate whether BCG or other forms of immunotherapy could be effective in the treatment of GBM.

Despite the uncertainty, this case raises important questions about the potential role of immunotherapy in the treatment of GBM and provides a rationale for further research in this area.

It is hoped that such research will contribute to the development of more effective therapies for GBM. Given the significant challenge that this aggressive cancer presents, it is crucial to explore all potential therapeutic avenues, including those that might seem unexpected. The potential immunomodulatory effects of BCG, a treatment traditionally associated with bladder cancer and tuberculosis, certainly fall within this category.

The immune system has long been known to play a crucial role in cancer development and progression but harnessing it effectively for cancer therapy has been a significant challenge [27]. The success of immunotherapies such as checkpoint inhibitors in various cancers has underscored the potential of this approach, and the possibility that BCG might have similar effects in GBM is an exciting prospect that warrants further investigation [31].

However, it is worth noting that the complex immunological environment of the brain and the immunosuppressive nature of GBM present specific challenges for immunotherapy in this context. GBM has various mechanisms to evade and suppress the immune response, including the production of immunosuppressive cytokines, the recruitment of regulatory T cells, and the expression of immune checkpoint molecules [32,33,34,35]. Overcoming these mechanisms will be a key challenge in the development of effective immunotherapies for GBM.

## 4. Conclusions

The intriguing case showcasing transient GBM regression after Bacillus Calmette–Guérin (BCG) therapy for bladder cancer prompts a critical need for a meticulous investigation into its underlying mechanisms and therapeutic prospects. While the precise mechanisms remain enigmatic, the potential systemic immunomodulatory effects of BCG on GBM warrant in-depth scrutiny. This scenario prompts pivotal inquiries into the complex interplay between systemic immune responses and the intricate microenvironment within GBM. An extensive exploration into the prospective immunomodulatory roles of BCG or analogous immunotherapies in GBM therapy holds significant promise. However, GBM’s innate immunosuppressive strategies, including cytokine secretion, regulatory T cell recruitment, and immune checkpoint expression, pose formidable challenges. The clinical implications are profound, suggesting a potential avenue for refining GBM treatment strategies by harnessing immune responses. Despite the success of analogous treatments in other cancers, overcoming GBM’s intrinsic immunosuppression necessitates rigorous investigation and innovative approaches. Interdisciplinary collaboration is imperative to elucidate the intricate dynamics between immunotherapy and GBM. Thorough preclinical investigations and meticulously designed clinical trials are imperative to ascertain the potential efficacy of BCG or similar immunotherapeutic interventions in reshaping GBM treatment paradigms. While this case signifies the intriguing potential of BCG-induced immunomodulation in GBM, comprehensive research is imperative. Successful exploration could offer new avenues for combatting this formidable disease, offering renewed optimism for individuals grappling with GBM’s challenges.

## Data Availability

No new data were created or analysed in this study.
